# Clinical translation of 3D bioprinting in oral and maxillofacial reconstruction: Recent progress and future directions

**DOI:** 10.1016/j.jobcr.2026.01.005

**Published:** 2026-01-30

**Authors:** Shantanu Dixit, Maher AL. Shayeb, Goma Kathayat, Dinesh Rokaya

**Affiliations:** a Dental Imaging & Diagnostic Center, Ballabgarh, Haryana, India; b Clinical Sciences Department, College of Dentistry, Ajman University, Ajman, United Arab Emirates; c Center of Medical and Bio-Allied Health Sciences Research, Ajman University, Ajman, United Arab Emirates; d Center of Excellence in Precision Medicine and Digital Health, Department of Physiology, Faculty of Dentistry, Chulalongkorn University, Bangkok 10330, Thailand

**Keywords:** 3D bioprinting, Oral and maxillofacial reconstruction, Bioinks, 4D bioprinting

## Abstract

**Background:**

Oral and maxillofacial reconstruction (OMF) requires regeneration of bone, soft tissue, vasculature, and nerves. Three-dimensional (3D) bioprinting offers a paradigm shift, enabling fabrication of patient-specific, cell-laden constructs designed to restore both anatomical form and biological function. This review presents an updated review on the clinical translation of 3D bioprinting in oral and maxillofacial reconstruction and presents future directions.

**Methods:**

A comprehensive literature search was conducted in PubMed, Scopus, Web of Science, and Google Scholar for studies published up to June 2025, using search terms such as “3D bioprinting,” “bioink,” “OMF reconstruction,” and tissue-specific phrases. Extracted data addressed bioprinting strategies, biomaterials, and outcomes, which were synthesized into translational phases and tissue-specific applications.

**Results:**

Four phases of translational progress were identified: (1) in vitro validation of bioinks and cell viability; (2) small-animal studies demonstrating osteogenesis, angiogenesis, and pulp–periodontal regeneration; (3) large-animal models addressing anatomical scalability and achieving partial functional integration; and (4) early human applications of acellular, patient-specific scaffolds. Success depends on tailoring bioinks—integrating stem cells, biomaterials, and signaling molecules—for tissues such as vascularized pulp, mineralized bone, and the periodontal ligament interface.

**Conclusion:**

3D bioprinting holds transformative potential for OMF reconstruction. While progress is evident from bench to large-animal studies, clinical adoption of viable, cell-laden constructs remains elusive. Overcoming biofabrication, integration, and regulatory challenges through interdisciplinary collaboration will be critical to realize the promise of patient-specific, functional bioprinted OMF tissues in clinical practice.

## Highlights

• 3D bioprinting enables patient-specific, functional constructs for oral and maxillofacial reconstruction.
• Strategies target alveolar bone, periodontium, gingiva, TMJ, and teeth, among other craniofacial tissues.
• Translation is limited by vascularization, neural integration, biomaterial standardization, and regulation.
• Emerging solutions include 4D bioprinting, organoids, neurovascular co-patterning, and AI integertaion.

## Graphical abstract

**Figure:**
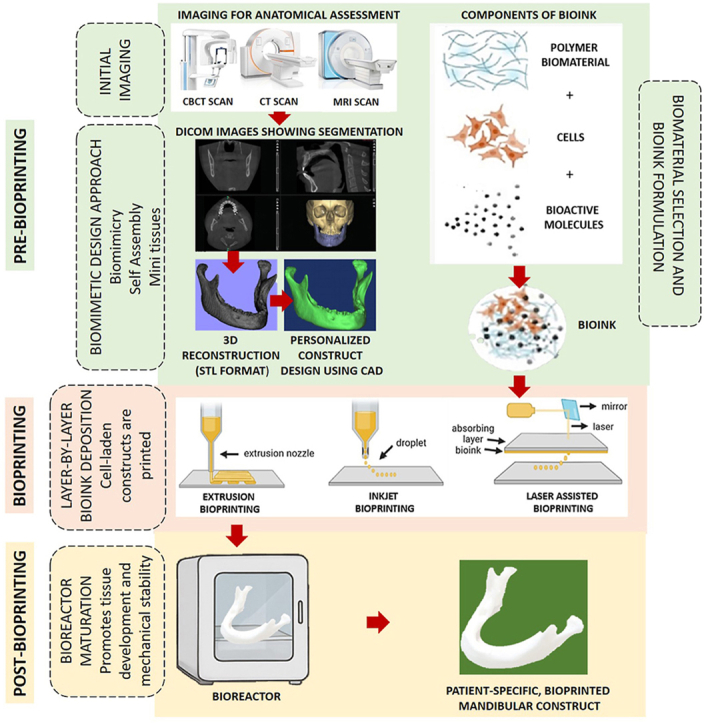

## 1 Introduction

### 1.1 Clinical challenges in OMF reconstruction

The OMF complex comprises anatomically and functionally interdependent structures, including cartilage, muscle, ligaments, vasculature, nerves, and the tooth–alveolar bone unit, that collectively maintain regional structural integrity and physiological balance.^1^ The prevalence of OMF defects is rising due to diverse etiologies, such as traumatic injuries (e.g., road traffic accidents, occupational or sports trauma), surgical resection of benign or malignant lesions, periodontal disease, odontogenic infections, congenital anomalies, and progressive atrophy from edentulism or systemic conditions.^2^^,^^3^ These conditions often result in significant hard and soft tissue loss or dysfunction. OMF impairment affects critical functions such as mastication, speech, and respiration, while also altering facial esthetics—factors that reduce quality of life and impact psychological and socioeconomic well-being.^4^^,^^5^

Reconstruction requires individualized, interdisciplinary planning to restore oral function and facial form.^6^ This is further complicated by the need to regenerate multiple, distinct tissue types—bone, musculature, mucosa, and neurovascular bundles—within anatomically constrained, esthetically sensitive, and biomechanically active regions.^1^ Therefore, successful OMF reconstruction demands more than anatomical restoration; it requires biologically integrated regeneration of form, function, and biomechanical harmony.^6^

### 1.2 Limitations of conventional grafting techniques

Grafting remains the mainstay of OMF reconstruction, employed to restore osseous and soft tissue structures.^2^ Autologous bone grafts remain the benchmark for hard tissue repair, given their inherent potential to support osteogenesis, osteoinduction, and osteoconduction.^7^ However, their application is limited by finite graft volume, donor site morbidity, prolonged surgical time, and increased postoperative discomfort.^8^ Although allogenic and xenogeneic grafts provide greater accessibility, they pose potential risks such as disease transmission, immunogenic reactions, and unpredictable remodeling.^9^ Synthetic bone substitutes offer safety and availability but are acellular and often fail to integrate predictably in biologically compromised environments.^3^

Soft tissue grafting similarly relies on autologous techniques, such as free gingival and subepithelial connective tissue grafts, which yield consistent results but are limited by donor tissue availability, procedural difficulty, and associated morbidity.^10^^,^^11^ Although biological substitutes exist, they are biologically inert and cannot replicate the microarchitecture or functional fidelity of native tissues. Typically supplied in standardized forms, they require intraoperative customization, complicating handling and increasing technique sensitivity.^12^ Collectively, these limitations highlight the need for next-generation regenerative strategies that are biomimetic, patient-specific, and capable of addressing the complex demands of OMF reconstruction.

### 1.3 Paradigm shift in OMF reconstruction: Transitioning from conventional grafting to 3D printing and bioprinting

In response to these limitations, progress in digital fabrication and regenerative engineering has introduced patient-tailored strategies for OMF reconstruction.^1^^,^^2^^,^^13^^,^^14^ 3D printing, commonly referred to as rapid prototyping, is a computer-guided process that constructs objects in successive layers from digital models.^15^^,^^16^ It allows the production of complex geometries that are challenging with conventional methods,^17^^,^^18^ offering exceptional dimensional accuracy for customized implants, guides, splints, and surgical instruments.^19^, ^20^, ^21^, ^22^ Consequently, 3D printing has gained clinical traction in OMF procedures, including segmental bone repair, orthognathic surgery, TMJ interventions, and scaffold fabrication to support tissue regeneration.^14^ In soft tissue applications, 3D-printed scaffolds offer superior anatomical fidelity and regenerative outcomes compared to conventional grafts.^23^^,^^24^

However, traditional 3D printing yields acellular constructs made of metals, polymers, or ceramics that lack biological cues essential for regeneration.^25^ These materials do not support cell viability, vascular ingrowth, or dynamic remodeling,^26^ limiting their utility in biologically demanding applications.^27^

This has spurred the emergence of 3D bioprinting, which integrates 3D printing technologies with tissue engineering principles.^13^ By incorporating viable cells, biomolecules, and bioactive matrices into layered structures, 3D bioprinting allows the generation of functional, tissue-like constructs that resemble the architecture and biochemical profile of their native counterparts.^28^, ^29^, ^30^, ^31^, ^32^.

A comparative overview of conventional 3D printing and bioprinting is provided in Table 1, highlighting their distinct features and clinical implications for OMF reconstruction.^1^^,^^2^^,^^13^^,^^14^^,^^25^^,^^33^^,^^34^

**Table 1:** Key differences between conventional 3D printing and bioprinting.

Parameter	Conventional 3D Printing	3D Bioprinting	References
Core Application	Fabrication of surgical guides, implant models, and anatomical replicas	Regeneration of living, functional tissue constructs tailored to patient-specific defects	^1^^,^^2^^,^^13^^,^^25^
Material Used	Inert materials (e.g., thermoplastics, metals, ceramics)	Bioinks composed of living cells, hydrogels, growth factors, and extracellular matrix (ECM) components	^1^^,^^2^^,^^13^^,^^25^
Biological Functionality	Acellular; does not support tissue growth or remodeling	Bioactive and cell-laden; enables proliferation, maturation, and host integration	^2^^,^^25^^,^^33^
Tissue Compatibility	Replicates geometry but lacks biological integration	Mimics biological, mechanical, and architectural features of native tissues	^2^^,^^25^^,^^33^
Soft Tissue Applications	Indirect support via molds or surgical templates	Direct fabrication of patient-specific soft tissues (e.g., gingiva, mucosa)	^1^^,^^2^^,^^14^
Vascularization Potential	Absent; no support for perfusion or angiogenesis	Actively explored; angiogenic bioinks and microvascular printing are under development	^1^^,^^25^
Customization Level	High geometric fidelity; limited to physical form	Anatomical and biological customization at cellular and structural levels	^13^^,^^14^^,^^25^
Surgical Integration	Used preoperatively for planning and implant positioning	Intended for intraoperative or prefabricated bioactive grafts	^13^^,^^14^
Regulatory & Clinical Readiness	Widely adopted with established standards	Emerging; mostly in preclinical or early-phase trials, with evolving regulatory frameworks	^2^^,^^14^^,^^34^
Limitations	Lacks biological responsiveness; unsuitable for regenerative applications	Requires advanced biofabrication protocols, cell sourcing, and bioink standardization	^1^^,^^25^^,^^33^
Clinical Impact & Future Potential	Enhances surgical accuracy and planning; confined to non-living constructs	Promises to transform personalized regenerative therapies through functional tissue grafting	^1^^,^^2^^,^^13^^,^^25^^,^^33^

### 1.4 Need for the review and its relevance to OMF reconstruction

3D bioprinting is reshaping tissue engineering, with accelerating clinical use in skin, cartilage, and bone regeneration. The global bioprinting market, valued at USD 2.13 billion in 2022, is projected to reach USD 8.3 billion by 2030, led by companies such as BICO and GE Healthcare.^35^ Despite this growth, OMF applications remain underdeveloped, hindered by anatomical complexity, high functional-aesthetic demands, and the need for precise, patient-specific reconstruction. While foundational reviews have addressed bioprinting in generalized or tissue-specific contexts, recent advances call for a renewed focus on OMF reconstruction—one that integrates bioink innovation, vascularization strategies, and regulatory progress into a unified translational framework. This review addresses that gap by tracing 3D bioprinting's progression from preclinical models to clinical adoption, analyzing OMF-specific strategies, and identifying key barriers to accelerate functional reconstruction.

## 2 Materials and methods

### 2.1 Literature search strategy

This article is a narrative review aimed at providing a comprehensive and expert synthesis of the current state of 3D bioprinting for OMF reconstruction. To enhance methodological transparency, we report our information sources, search strategy, and approach to data synthesis in alignment with key principles of the PRISMA-ScR (Preferred Reporting Items for Systematic Reviews and Meta-Analyses extension for Scoping Reviews) framework, without claiming full scoping-review methodology.

A structured literature search was conducted to identify publications pertaining to 3D bioprinting and its application in OMF reconstruction. Electronic databases, including PubMed, Scopus, Web of Science, and Google Scholar, were queried for studies published up to June 2025. To capture the most recent technological advancements, emphasis was placed on literature from the past decade (2015–2025). Seminal works published prior to this period were also incorporated to provide essential historical context and foundational insights into scaffolds, stem cell biology, and additive manufacturing.

The search strategy employed a combination of Medical Subject Headings (MeSH) terms and free-text keywords (e.g., “3D bioprinting”, “biofabrication”, “additive manufacturing”, “oral and maxillofacial reconstruction”, “craniofacial tissue engineering”, “bone”, “enamel”, “dentin”, “dental pulp”, “dentin-pulp complex”, “periodontium”, “temporomandibular joint”, “bioinks”, “stem cells”), combined with Boolean operators (AND, OR) to refine results. Article selection was finalized through an expert-guided, iterative process, including review of reference lists of key papers to ensure comprehensive coverage of the field.

### 2.2 Study selection and eligibility criteria

Inclusion criteria encompassed: (i) original research (in vitro, in vivo, or clinical), (ii) systematic reviews, meta-analyses, or influential perspective articles addressing 3D bioprinting in OMF regeneration, and (iii) direct translational or clinical relevance to OMF reconstruction.

Exclusion criteria included: (i) non-English publications, (ii) conference abstracts without accessible full text, (iii) grey literature, and (iv) duplicate studies. Articles focused solely on general bioengineering principles without explicit dental or maxillofacial applications were not prioritized.

### 2.3 Data extraction and synthesis

All identified references were imported into EndNote (Clarivate Analytics) for management, enabling duplicate removal and structured organization of the literature library. Data extraction emphasized study objectives, bioprinting methodologies (including cell sources, bioink composition, and printing techniques), key findings, and translational potential.

The extracted data were synthesized thematically into the following domains: (i) foundational principles and key components of 3D bioprinting, (ii) the evolutionary trajectory of bioprinting in OMF reconstruction, from in vitro models to early clinical applications, (iii) customization of bioprinting strategies for specific OMF tissues, and (iv) persistent translational barriers and emerging future directions.

## 3 3D bioprinting and key components

With the ongoing advancement of 3D bioprinting in regenerative medicine, it is critical to examine its fundamental components and design strategies to unlock its clinical applications in OMF reconstruction.

### 3.1 3D bioprinting

3D bioprinting is defined as the layer-by-layer, spatially controlled deposition of biological materials, biochemicals, and living cells to fabricate functional, three-dimensional structures.^25^ It is an automated, computer-aided design (CAD)-driven process that differs from conventional additive manufacturing by its capacity to co-deposit biomaterials and living cells in a predesigned spatial architecture. This capability enables the creation of geometrically complex, cellularized constructs and has broad applications across biomedicine, with specific relevance to OMF tissue reconstruction.^13^^,^^36^

### 3.2 Central design approaches for 3D bioprinting

Biomimicry, self-assembly, and mini-tissue fabrication represent foundational strategies driving bioprinting innovation. These approaches aim not only to replicate tissue structure but also to promote biological functionality and integration of bioprinted constructs.^25^^,^^37^

Biomimicry seeks to replicate the cellular, extracellular, and biomechanical characteristics of native tissues. This requires a detailed understanding of cell-type organization, bioactive gradient distribution, ECM composition, and local mechanical forces.^25^^,^^38^^,^^39^ Advances in multi-material printing have improved the capacity to fabricate highly biomimetic constructs.^37^

Self-assembly leverages the innate capacity of cells to organize and differentiate without external scaffolds, mimicking embryonic tissue development. Cells secrete ECM, initiate signaling cascades, and undergo spatial patterning to form microarchitectures reflective of native tissues.^25^^,^^37^^,^^40^^,^^41^ Scaffold-free methods, including spheroid bioprinting, enhance biological fidelity by enabling high cell–cell interaction.^42^^,^^43^

**Fig. 1:**
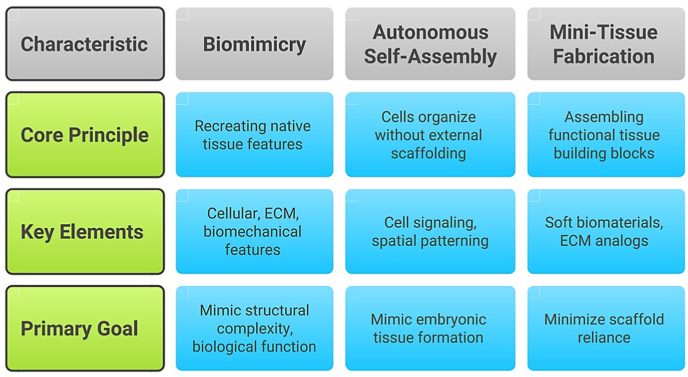
Overview of the central design strategies driving 3D bioprinting.

Mini-tissue strategies integrate elements of biomimicry and self-assembly by constructing small, functional tissue units that can be assembled into larger constructs.^25^^,^^37^^,^^44^ These units rely on soft biomaterials and ECM analogs—such as hydrogels—to support maturation, reduce scaffold dependence, and promote integration.^44^, ^45^, ^46^ Fig. 1 summarizes how each of these biological strategies contributes to the design and fabrication of living tissue through bioprinting.

### 3.3 Key components of 3D bioprinting

The performance of 3D bioprinting depends on the precise coordination of core components, each of which contributes to the construct's anatomical fidelity, biological viability, and translational relevance.

#### 3.3.1 Design and imaging

Accurate bioprinting begins with high-resolution imaging and 3D modeling. Modalities such as computed tomography (CT), cone beam CT (CBCT), and magnetic resonance imaging (MRI) provide noninvasive visualization of anatomical structures at various scales.^25^ CT offers high-resolution cross-sectional imaging via X-ray absorption,^47^ while CBCT delivers fast acquisition, lower radiation, and excellent 3D imaging of hard tissues—particularly useful in maxillofacial applications.^48^ MRI offers excellent soft tissue differentiation while avoiding exposure to ionizing radiation, and its performance can be further improved with contrast agents such as gadolinium or iron oxide.^25^^,^^49^^,^^50^

These imaging datasets are reconstructed into 3D anatomical models and refined using CAD tools and mathematical modeling to generate print-ready constructs.^51^^,^^52^ Depending on the clinical objective, the design may replicate native anatomy or incorporate tailored geometries.^53^ The final model is sliced into 2D digital layers, guiding the layer-by-layer printing process. Optimal design alignment with printer resolution is critical to achieving accurate anatomical and functional replication.^53^, ^54^, ^55^, ^56^.

#### 3.3.2 Bioink

Bioinks are cell-containing formulations engineered for automated bioprinting, often blended with biomaterials and bioactive molecules. They may consist of single cells, spheroids, mini-tissues, or organoids embedded in hydrogels or microcarriers.^2^ Additional elements, such as growth factors, miRNA, DNA, or exosomes, may be incorporated depending on the application.^57^

**Fig. 2:**
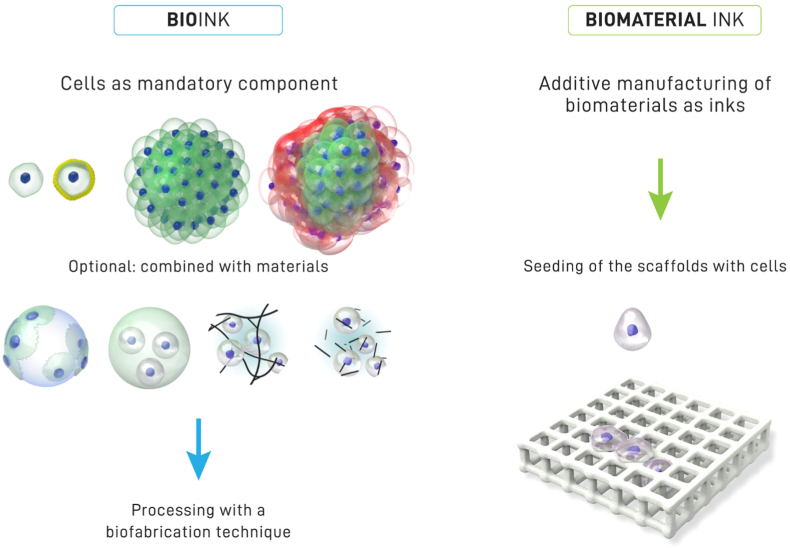
Schematic illustrating the distinction between bioinks and biomaterial inks: bioinks incorporate cells as a mandatory component during fabrication, whereas biomaterial inks involve cell seeding after scaffold printing. Adapted from ref. (58) distributed under Creative Commons Attribution License (CC BY 4.0).

A distinction is made between bioinks, which contain cells during fabrication, and biomaterial inks, which are cell-free at the time of printing, with cells added post-fabrication.^58^ Fig. 2 illustrates this conceptual difference. The functionality of a bioink is defined by three principal components—seed cells, biomaterials, and bioactive molecules—each discussed below.

##### 3.3.2.1 Seed cells

Seed cells form the biological foundation of bioinks, playing a pivotal role in maintaining construct architecture and driving regenerative outcomes.^59^ Ideal characteristics include high printability, proliferation, differentiation capacity, genetic stability, and scalability.^60^ Early studies relied on differentiated or immortalized cell lines, but mesenchymal stem cells (MSCs) have become the preferred source because of their multipotent capacity, immunomodulatory effects, and minimal immunogenicity.^59^^,^^61^^,^^62^

MSCs are broadly classified into systemic such as bone marrow-derived mesenchymal stem cells (BMSCs), adipose-derived stem cells (ADSCs), and umbilical cord-derived MSCs (UC-MSCs) and oral-derived subtypes, including dental pulp stem cells (DPSCs), stem cells from human exfoliated deciduous teeth (SHED), and periodontal ligament stem cells (PDLSCs). Systemic MSCs are easily harvested and have shown regenerative success in bone and soft tissues but may require inductive cues for specificity in the OMF region.^63^, ^64^, ^65^.

Oral-derived MSCs, derived from the neural crest, offer site-specific regenerative potential: DPSCs and SHED promote pulp–dentin regeneration and angiogenesis^66^, ^67^, ^68^, ^69^, while PDLSCs, stem cells from the apical papilla (SCAPs), dental follicle stem cells (DFSCs), and gingival mesenchymal stem cells (GMSCs) support regeneration of gingiva, periodontal ligament, and root structures.^67^^,^^70^, ^71^, ^72^, ^73^ Buccal fat pad-derived stem cells (BFPSCs) and alveolar-derived skeletal stem/progenitor cells (SSPCs) show promise in alveolar bone regeneration.^74^, ^75^, ^76^.

Pluripotent stem cells—including induced pluripotent stem cells (iPSCs) and human embryonic stem cells (hESCs)—offer broader differentiation capacity. iPSCs from dental or non-dental sources have been applied in pulp, periodontal, and TMJ regeneration, though risks of tumorigenicity and genomic instability persist.^59^^,^^61^^,^^77^, ^78^, ^79^, ^80^, ^81^ hESCs, while highly potent, raise ethical and immunologic concerns, limiting their translational use in OMF contexts.^61^^,^^80^^,^^81^

Scaffold-free strategies using self-assembled spheroids from DPSCs, SCAPs, or SHED are emerging to overcome scaffold-related limitations. These constructs allow dense cell–cell interaction and support vascularized pulp–dentin regeneration, although structural support with hydrogels is often necessary.^61^^,^^82^, ^83^, ^84^, ^85^, ^86^ Table 2 compares representative seed cell types in terms of their biological features and applications in OMF bioprinting.

**Table 2:** Summary of key bioink components: Seed Cells, Biomaterials, and Bioactive Molecules, along with their ideal properties.

	Representative Seed Cells Incorporated in Bioink					
	Cell Type	Source	Advantages	Limitations	OMF Applications	
Seed Cells	**MSCs (systemic)**	Bone marrow, adipose tissue, umbilical cord	Easy harvest; immunomodulatory; low immunogenicity; BMSCs are osteogenic; ADSCs support pulp/PDL lineages; UC-MSCs are easily harvested	Lower multipotency than ESCs; variable differentiation efficiency	Alveolar bone repair (BMSCs); potential for pulp and periodontal regeneration (ADSCs, UC-MSCs); TMJ disc regeneration (UC-MSCs)	^59^^,^^61^, ^62^, ^63^^,^^77^
	**MSCs (Oral derived)**					
	DPSCs	Pulp of unerupted third molars	High angiogenic potential; Forms pulp-dentin complex; Multipotent differentiation (odontoblasts, neurogenic, angiogenic)	Requires optimal scaffold/microenvironment; Sensitive to oxygen tension and matrix stiffness	Dentin-pulp complex formation, dentin repair, bone formation; potential for neurovascular regeneration; whole tooth regeneration; TMJ disc regeneration	64, 65, 66^,^^68^^,^^77^
	SHED	Exfoliated deciduous teeth	Easily obtainable; High proliferative, angiogenic, osteoinductive and endothelial differentiation capabilities; Suitable for pediatric use	Limited to pediatric donors; Inter-donor variability	Dentin- and pulp-like tissue formation; Angiogenesis; Osteoinduction	^64^^,^^65^^,^^67^^,^^69^
	PDLSCs	Periodontal ligament	Promotes PDL and cementum regeneration; Induces angiogenesis; Easy access during extraction	Lower osteoinductive potential than DPSCs/SHED; Heterogeneity in cell populations	Periodontal tissue regeneration; Clinical safety in pilot studies; whole tooth regeneration; TMJ disc regeneration	64, 65, 66^,^^70^^,^^77^
	SCAPs	Apical papilla of immature teeth	High proliferative and mineralization capacity; Promotes root maturation and dentinogenesis	Availability limited to immature teeth; RET outcomes inconsistent	Root development and dentin regeneration; Used in regenerative endodontics; whole tooth regeneration	64, 65, 66^,^^71^
	DFSCs	Dental follicle tissue	Potential for odontogenic, root, and periodontal differentiation	Less studied than DPSCs/SHED; Requires tooth germ availability	Regeneration of dentin, root, and periodontal structures	^64^^,^^65^^,^^72^
	GMSCs	Gingival connective tissue	Osteogenic potential; Non-invasive harvesting; Supports PDL regeneration	In vivo application still under early investigation	Periodontal ligament regeneration; Treatment of gingival lesions	^64^^,^^65^^,^^73^
	BFPSCs	Buccal fat pad	Good osteogenic differentiation; Proximity to OMF site	Limited data in OMF applications	Potential for alveolar bone regeneration	^64^^,^^65^^,^^74^
	Alveolar BMSCs/SSCs/SSPCs^a^	Alveolar bone, periosteum	Strong osteogenic lineage; Expresses osteogenic markers (ALP, RUNX2, OCN, OPN); Periosteum-derived cells are accessible	Invasive harvest; Morbidity associated with donor site	Excellent for alveolar bone regeneration, especially from mandibular SSPCs	^65^^,^^75^^,^^76^^,^^83^
	**iPSCs**	Reprogrammed adult somatic cells: Dental (DPSCs, SHED, PDL fibroblasts); Non dental (skin fibroblasts, blood cells)	High pluripotency; no ethical issues; autologous; expandable in vitro	Risk of tumorigenesis; genetic instability; low reprogramming efficiency	Bone and pulp-dentin regeneration, PDL repair, whole-tooth engineering; TMJ disc regeneration	^61^^,^^77^, ^78^, ^79^
	**hESCs**	Inner cell mass of blastocysts	High pluripotency; differentiates into all tissues	Ethical concerns; risk of immune rejection and tumorigenicity	Experimental enamel/dentin regeneration; early-stage whole-tooth engineering	^61^^,^^80^^,^^81^
	**Scaffold-Free Cellular Aggregates**	Self-assembled spheroids (e.g. DPSCs, SCAPs, SHED, HERS cells)	High cell density; avoids scaffold toxicity	Needs hydrogel encapsulation for mechanical support	Dental pulp regeneration with neurovascularized dentin-pulp complex	^45^^,^^61^^,^^84^, ^85^, ^86^

	Commonly Used Biomaterials (Polymers) in Bioink									
	Type	Polymer Name	Water Solubility	Cross-linking Method	Print-ability	Bio-compatibility	Merits	Demerits	OMF Reconstruction Benefits/Applications(Examples)	
**Biomaterials**	Natural	Agarose	Yes	Thermal/Ionic	High	Moderate	High mechanical strength, low cost, quick gelation	Inferior cell adhesion, requires blending for functionality	Alveolar bone bioprinting, TMJ disc scaffolds	^77^^,^^90^, ^91^, ^92^, ^93^, ^94^
		Alginate	Yes	Ionic(e.g., Ca^2+^)	High	Moderate	Biocompatible, low cost, tunable viscosity	Low cell adhesion, risk of nozzle clogging	Vascularized pulp-dentin constructs; odontoblast differentiation with dentin matrix, TMJ cartilage blends (with collagen)	^77^^,^^90^^,^^93^, ^94^, ^95^, ^96^, ^97^
		Collagen	pH-dependent	pH/temperature/enzymatic	Moderate	High	Enhances cell adhesion, mimics native ECM	Slow gelation, low mechanical stability, high cost	DPSC viability (>95 %); capillary network formation with HUVECs, TMJ disc/cartilage (hybrid scaffolds)	^77^^,^^90^^,^^93^^,^^97^, ^98^, ^99^
		HyA	Yes	Chemical (meth--acrylate)/Photo-crosslinking	Moderate	High	Promotes cell growth, angiogenesis, biocompatible	Fast degradation, low mechanical properties	Angiogenic bone/pulp scaffolds, cartilage ECM mimic	^77^^,^^90^^,^^97^^,^^100^, ^101^, ^102^
		Fibrin	Yes	Enzymatic (thrombin)	Moderate	High	Supports angiogenesis, rapid gelation	Weak mechanical strength, requires thrombin for crosslinking	Pulp-dentin regeneration, chondrocyte encapsulation	^77^^,^^90^^,^^97^^,^^100^, ^101^, ^102^
		CMC	Yes	Thermal	Moderate	Moderate	Biocompatible, good mechanical integrity	Requires chemical modification for enhanced functionality	Mimics native ECM for pulp-dentin and periodontal tissues	^90^^,^^103^^,^^111^^,^^128^^,^^131^^,^^132^
		Silk fibroin	Partial	Thermal/Chemical	Moderate	Moderate	High mechanical strength, biocompatible	Low cell viability without additives, complex processing	Bone mineralization, TMJ disc reinforcement	^77^^,^^104^, ^105^, ^106^, ^107^, ^108^, ^109^, ^110^
		dECM	Variable	Thermal/pH/Photo-crosslinking	Moderate	High	High biocompatibility, preserves native ECM components	Complex and costly isolation process, limited availability	Pulp-dentin/periodontal tissues, TMJ disc regeneration	^77^^,^^90^^,^^112^, ^113^, ^114^, ^115^, ^116^
		GelMA	Yes	Photo-crosslinking (UV/visible light)	Moderate-High	High	Tunable mechanical properties, ECM-mimetic, biodegradable	Low mechanical strength, fast degradation, UV crosslinking may be cytotoxicity	DPSC encapsulation (>80 % viability); vascularized alveolar bone with PCL support	^90^^,^^117^
	Synthetic	Pluronic®	Yes	Photo-polymerization	High	Moderate	Reversible gelation, shear-thinning properties	Low mechanical stability, fast degradation	Sacrificial material for precision in alveolar bone bioprinting	^90^^,^^93^^,^^96^^,^^97^^,^^118^
		PEG/PEO	Yes	Photo-polymerization	High	Moderate	Tunable mechanical properties, biocompatible	Low cell adhesion, UV curing may damage cells	Mechanical reinforcement in fibrin/PEG pulp-dentin scaffolds	^77^^,^^90^^,^^97^
		PCL	No	None (Thermo-plastic) or UV/Chemical	High	Moderate-High	Slow hydrolytic degradation, High strength, Thermally stable (liquid phase), Bioceramic-compatible	Hydrophobic Slow degradation, Non-osteogenic, Requires post-printing cell seeding	Mechanical support for GelMA in alveolar bone, TMJ disc (with chitosan)	^77^^,^^90^^,^^119^^,^^120^
	Inorganic	**Metals**								
		Ti	Insoluble	None	High	Excellent	High strength, osseointegration, corrosion resistance	Non-degradable, potential stress shielding	Personalized craniofacial and mandibular implants	^2^^,^^121^
		MgO	Slightly soluble	Ionic bonding	Medium	Excellent	Promotes osteogenesis, antibacterial, biodegradable	Rapid degradation, requires composite use	Bone scaffolds, antibacterial OMF materials	^2^^,^^122^
		Fe/Mn	Insoluble	None	High	Good	Bone-mimicking elasticity, printable porous structure, degradable	Slow resorption (Fe), complex optimization	Porous biodegradable scaffolds, OMF implants	^2^^,^^123^
		**Bioceramic materials**								
		HA	Insoluble	None	High	Good	Bone-like mineral, osteoconductivity, bioactivity	Brittle, slow degradation	Bone regeneration, dental and hard tissue repair	^2^^,^^124^
		β-TCP	Slightly soluble	None	High	Good	Resorbable, osteoconductive	Low mechanical strength, brittleness	Bone scaffolds, maxillofacial defect filling	^2^^,^^125^
		BCP	Insoluble	None	High	Good	Balanced bioactivity and resorption	Brittle	Dental and craniofacial bone engineering	^2^^,^^126^
		Bioactive glass	Insoluble	None	Medium	Excellent	Ionic release supports bone bonding and regeneration	Brittle, slow degradation	Bone defect repair, scaffold coatings for OMF	^2^^,^^127^
	Composite	Natural-Natural/Synthetic/Inorganic Blends	Variable	Depends on components	High (if polymer-based)	High (if biocompatible components)	Synergistic properties	Requires optimization of ratios/blending	Refer Table 4 for formulations and outcomes	^2^^,^^33^^,^^77^^,^^90^^,^^129^^,^^130^

	Bioactive Molecules Used in Bioinks				
	Types	Bioactive Component	Biomaterial (Polymers)	Applications	
**Bioactive Molecules**	Growth factor	BMP-2	GelMA and collagen; Alginate, collagen, gelatin, PCL; Collagen, gelatin, PCL, PLGA; HA, PEG, PLGA; Alginate, alginate sulfate; Gelatin micro-particles in alginate	Bone formation	^135^^,^^137^, ^138^, ^139^, ^140^, ^141^
		TGF-β	Alginate, PCL; PLGA micro-particles in PCL; HA, polyurethane; PLGA Nanoparticles in GelMA	Cartilage formation	142, 143, 144, 145
		BMP-7	PLGA micro-particles in PCL	Dental tissue formation	^146^
		NGF	PLGA Nanoparticles in PEG	Neural tissue formation	^147^
		VEGF	Collagen, fibrin	Vascularization	^149^
			GelMA	Skeletal muscle injuries	^150^
		NGF	Silk Fibroin/Collagen	Nerve formation	^148^
	Enzyme	Tyrosinase	GelMA and Collagen	Skin formation	^151^
	DNA	pDNA	RGD-γ-irradiated alginate nHA	Bone formation	^152^
		Polypeptide-DNA	Hydrogel	Cartilage formation	^153^

**ADSCs:** Adipose-Derived Stem Cells**; β-TCP:** beta-tricalcium phosphate; **BCP:** Biphasic Calcium Phosphate; **BMP:** Bone morphogenetic protein-2; **BMSCs:** Bone Marrow Stem Cells; **BFPSCs:** Buccal Fat Pad Stem Cells; **CMC:** Carboxy-Methyl Cellulose; **dECM**: Decellularized ECM; **DPSCs:** Dental Pulp Stem Cells; **DFSCs**: Dental Follicle Stem Cells; **ECM:** Extracellular matrix; **Fe:** Iron; **GelMA:** Gelatin Methacrylamide; **GMSCs:** Gingival Mesenchymal Stem Cells; **HA:** Hydroxyapatite; **HERS**: Hertwig's Epithelial Root Sheath; **hESCs:** Human Embryonic Stem Cells; **HUVECs:** human umbilical vein endothelial cells; **HyA:** Hyaluronic Acid; **iPSCs:** Induced Pluripotent Stem Cells; **Mn:** Manganese; **MgO:** Magnesium Oxide; **MSCs:** Mesenchymal Stem Cells; **nHA:** nano-hydroxyapatite: **NGF:** Nerve growth factor; pDNA: Plasmid DNA; **PEG:** Poly(ethylene glycol); **PEO:** poly(ethylene oxide); **PCL:** Poly - (ε-caprolactone); **PDLSCs:** Periodontal Ligament Stem Cells; **PLGA:** Poly(D, L-lactic-co-glycolic acid); **SCAPs:** Stem Cells from Apical Papilla; **SHED:** Stem Cells from Human Exfoliated Deciduous Teeth; **TGF-β:** Transforming Growth Factor-Β; **Ti:** Titanium; **UC-MSCs:** Umbilical Cord-Derived MSCs; **VEGF:** Vascular Endothelial Growth Factor.

a Collectively refers to mesenchymal and skeletal stem/progenitor cells derived from alveolar bone; subsets may vary by study.

##### 3.3.2.2 Biomaterials

Biomaterials, whether derived from synthetic or natural sources, are non-pharmaceutical entities designed to temporarily or permanently support, restore, or replace damaged tissues and organs.^87^ In 3D bioprinting, they serve as scaffolding matrices, enabling cell encapsulation, spatial patterning, and biochemical signaling.^13^^,^^25^ By performing functions analogous to the extracellular matrix (ECM), they help sustain cellular viability, promote adhesion, and direct lineage-specific differentiation.^13^

Key characteristics of bioink-compatible biomaterials include printability, biocompatibility, biodegradability, ECM mimicry, and sufficient mechanical stability to support post-printing maturation and remodeling.^25^^,^^88^ While this section focuses on scaffold-based systems, scaffold-free alternatives using cell aggregates are discussed in subsequent sections.^89^

Biomaterials are generally categorized as natural, synthetic, inorganic, or composite.^1^^,^^2^^,^^90^ Natural polymers (e.g., alginate, agarose, collagen, hyaluronic acid, fibrin, CMC, silk fibroin) offer excellent biocompatibility but often lack mechanical strength, requiring reinforcement for shape fidelity.^91^, ^92^, ^93^, ^94^, ^95^, ^96^, ^97^, ^98^, ^99^, ^100^, ^101^, ^102^, ^103^, ^104^, ^105^, ^106^, ^107^, ^108^, ^109^, ^110^, ^111^ Decellularized ECM (dECM) bioinks are especially promising for pulp–dentin and periodontal regeneration 112, 113, 114, 115, 116, while gelatin methacryloyl (GelMA), a photo-crosslinkable ECM mimic, enables tunable crosslinking and has been used in vascularized alveolar scaffolds.^117^ Synthetic polymers (e.g., Pluronic®, PEG/PEO, PCL) offer precise control over rheology and mechanical behavior but require surface modification to promote biological activity.^1^^,^^2^^,^^33^^,^^90^^,^^118^, ^119^, ^120^ Inorganic biomaterials (e.g., titanium, MgO, Fe/Mn, HA, β-TCP, BCP, bioactive glass) provide osteoconductivity and mechanical support, making them suitable for alveolar, mandibular, and TMJ applications.^2^^,^^119^^,^^121^, ^122^, ^123^, ^124^, ^125^, ^126^, ^127^, ^128^ Composites integrate properties of natural, synthetic, or inorganic materials to enhance performance. For instance, alginate–HA blends improve osteogenesis, while PCL–gelatin hybrids improve both strength and biocompatibility.^2^^,^^33^^,^^77^^,^^90^^,^^129^, ^130^, ^131^, ^132^.

Table 2 presents a comparative overview of these biomaterials, emphasizing their physicochemical properties, bio-functionality, and suitability for various OMF applications in 3D bioprinting. While this list provides a foundational reference for key materials in the field, it does not encompass the full range of biomaterials currently under investigation —an area that continues to evolve with ongoing advances in regenerative bioprinting.

##### 3.3.2.3 Biomolecules/bioactive molecules

Bioactive molecules are critical components of bioinks, added to influence essential cellular processes including motility, growth, lineage commitment, and ECM remodeling, all of which are fundamental to tissue regeneration. These biologically active agents include growth factors, enzymes, and nucleic acid-based materials, all of which modulate the cellular microenvironment within printed constructs.^133^, ^134^, ^135^.

Their delivery is typically achieved through blending within hydrogels, entrapment in microparticles or nanoparticles, or covalent tethering to scaffold materials—enabling localized and temporally controlled release while preserving bioactivity.^57^^,^^136^^,^^137^ Such strategies are essential to replicate the spatial and temporal gradients of signaling molecules observed during native tissue development and repair.^57^

Among protein-based growth factors, bone morphogenetic protein-2 (BMP-2) is frequently applied to promote osteogenesis and has been incorporated into various biomaterial platforms, including natural polymers such as gelatin methacryloyl (GelMA) and collagen, polysaccharides like alginate, and synthetic polymers such as polycaprolactone (PCL) and poly(lactic-co-glycolic acid) (PLGA).^135^^,^^137^, ^138^, ^139^, ^140^, ^141^ Transforming growth factor-beta (TGF-β) supports chondrogenesis and cartilage formation when integrated into alginate, PLGA, polyurethane, or GelMA matrices.^142^, ^143^, ^144^, ^145^ BMP-7, another osteoinductive factor, has shown potential in dental tissue engineering when delivered via PLGA microparticles embedded in PCL scaffolds.^146^ In neural tissue engineering, nerve growth factor (NGF) encapsulated in PEG or silk fibroin–collagen matrices enhances axonal regeneration and neural tissue integration.^147^^,^^148^ Vascular endothelial growth factor (VEGF), widely studied for angiogenesis, is commonly incorporated into collagen, fibrin, or GelMA-based systems.^149^^,^^150^

Beyond protein growth factors, enzymes such as tyrosinase have been incorporated into GelMA and collagen-based bioinks to promote skin regeneration through oxidative crosslinking and pigmentation pathways.^151^ Nucleic acid-based agents like plasmid DNA (pDNA), often incorporated into RGD-modified, γ-irradiated alginate and nano-hydroxyapatite (nHA) constructs, have demonstrated potential for localized osteogenic gene expression.^152^^,^^153^

Table 2 provides a general overview of these bioactive molecules, their biomaterial carriers, and tissue targets. Their OMF-specific applications, including targeted combinations and translational models, are discussed in detail in the subsequent sections reviewing tissue-specific strategies and translational phases.

#### 3.3.3 Bioprinting strategies

Bioprinting strategies for OMF reconstruction have evolved from early platforms, including inkjet printing, extrusion systems, and laser-assisted methods, toward next-generation technologies capable of fabricating zonal, vascularized, and multi-tissue constructs.^25^^,^^37^^,^^61^ Each strategy is governed by distinct physical principles and imposes specific requirements on bioink characteristics, including viscosity, gelation kinetics, and surface tension, which in turn influence spatial fidelity, cell viability, and structural stability.^77^^,^^154^^,^^155^

In inkjet bioprinting, thermal or piezoelectric actuation generates pulses that dispense low viscosity bioinks in the form of individual droplets. This method enables rapid cell patterning but provides limited mechanical support, making it more appropriate for soft tissue applications such as gingiva and periodontal ligament.^156^^,^^157^ Extrusion-based bioprinting, driven by pneumatic or mechanical pressure, accommodates a broad viscosity range and allows multi-material deposition. Its capacity for higher-volume output and enhanced structural integrity makes it suitable for fabricating constructs for bone, pulp–dentin, and temporomandibular joint (TMJ) regeneration.^158^, ^159^, ^160^ Laser-assisted approaches, particularly laser-induced forward transfer (LIFT), enable precise patterning with excellent cellular survival and circumvent problems associated with nozzle-based printing. However, its operational complexity and cost limit clinical scalability; it is mainly explored for microvascular and hard tissue applications.^161^^,^^162^

Selecting an appropriate bioprinting strategy for OMF reconstruction requires balancing precision, bioink compatibility, and biological function. For instance, multi-material extrusion platforms have been used to fabricate osteochondral TMJ constructs,^143^ while coaxial and light-assisted systems have enabled vascularized pulp and periodontal ligament regeneration.^163^^,^^164^ Recent innovations include multi-head deposition systems (MHDS) and hybrid platforms that integrate multiple printing modalities to achieve higher functional complexity. Additionally, 4D bioprinting—designed to enable dynamic shape recovery and time-dependent adaptation—has garnered attention, although its clinical translation in dentistry remains preliminary.^61^^,^^155^^,^^165^ Table 3 presents a comparative overview of the operational parameters of the three principal bioprinting modalities. Table 4 highlights representative studies demonstrating their tissue-specific applications across various stages of clinical translation in OMF reconstruction.

**Table 3:** Parameter-based evaluation of bioprinting systems.

Parameter	Description	Inkjet-Based System	Extrusion-Based System	Laser-Based System	Reference
**Mechanism-Related Parameters**					^25^^,^^37^^,^^61^^,^^77^^,^^154^^,^^155^
**Working Principle**	Mechanism enabling layer-by-layer bioink deposition	Based upon droplet ejection via thermal/piezoelectric	Based upon continuous filament extrusion under pressure	Based upon Laser-induced forward transfer (LIFT)	
**Subtypes**	Specific types within each system	Thermal inkjet, Piezoelectric inkjet, Acoustic droplet ejection	Embedded, Co-axial, Single-/multi-nozzle, Continuous chaotic printing	LIFT	
**Actuation Method**	Force mechanism for material deposition	Thermal/piezoelectric	Pneumatic, piston, or screw	Laser pulse or optical trapping	
**Bioink-Related Parameters**					
**Viscosity Range**	Range of bioink viscosity compatible	<10 mPa s	30–6 × 10^7^ mPa s	1–300 mPa s	
**Gelation Method**	Method by which bioink solidifies	Chemical, photo-crosslinking	Chemical, photo-crosslinking, sheer thinning, temperature	Chemical, photo-crosslinking	
**Material Compatibility**	Types of printable biomaterials	Low-viscosity inks, hydrogels	Broad polymer and hydrogel range	Photosensitive hydrogels, ECM	
**Biological Parameters**					
**Cell Viability**	Percentage of live cells post-printing	>85 %	80 %–90 %	>95 %	
**Cell Density**	Number of cells per mL printable	Low <10^6^ cells ml^−1^	High, cell spheroids	Medium (10^8^ cells ml^−1^)	
**Single-Cell Resolution**	Ability to position or isolate single cells	Low	Medium	Medium	
**Technical & Operational Parameters**					
**Printer Cost**	Relative cost of the system	Low	Medium	High	
**Preparation Time**	Time needed for setup and calibration	Low	Low to medium	Medium to high	
**Output & Application Parameters**					
**Printing Resolution**	Minimum feature size achievable	10–50 μm	200–1000 μm	10–100 μm	
**Printing Speed**	Speed of bioink deposition	Fast (1–10,000 droplets per second)	Slow (10–50 μm/s)	Medium-fast (200–1600 mm/s)	
**Build Volume**	Maximum construct size	Small to moderate	Large	Small	
**Structural Integrity**	Mechanical stability of printed constructs	Poor	Good	Excellent	
**Multi-Material Capability**	Ability to print multiple materials simultaneously	Limited	Good	Moderate	
**Target Applications**	General clinical and biomedical applications	Tissue Engineering, Cell Patterning, Biological Component Delivery	Tissue Regeneration, Organ Models, Cell-Laden Structures	High Precision Constructs, Durable Tissue Models, Advanced Biomedical Research	
**Advantages**		Fast, low-cost, good for patterning, easy availability	Broad bioink compatibility, affordability, rapid printing, and multi-material capability	High precision and resolution, ability to print high-viscosity bio-ink, no nozzle clogging	
**Disadvantages**		Prone to clogging, limited to low viscosity, lacks precision	Lower resolution, limited to thermoplastic materials only, shear stress may affect viability, cell embedding limitation	High cost, time consuming, complex setup, Photocrosslinker toxicity

**Table 4:** Summary of key studies demonstrating the evolution (by phase) and application-specific customization of 3D bioprinting strategies in OMF tissue reconstruction.

Evolution phase	Target Tissue	Model	Material Type	Bioink/Bioink Material			Fabrication Approach/Cell Incorporation Strategy	Bio-printing Technique	Outcome	Reference
				Scaffold material	Bio-active material	Cell Type(s)				
Phase 1: Laboratory phase (In Vitro/Scaffold Only)	Cranio-facial bone	In vitro	Bioink	Alginate + gelatin + nHAp	–	hPDLSCs	Bioprinted construct/Embedded	Extrusion	High cell viability, adhesion, and osteogenic potential in bioprinted scaffolds	^158^
		In vitro	Bioink	GelMA + kCA + nSi (NICE bioink)		hMSCs	Bioprinted construct/Embedded	Extrusion	Mechanically robust, calcium-rich bone-like structures with cell remodeling	^176^
		In vitro	Bioink	GelMA + HAMA + LAP/type I collagen	–	IDG-SW3 (osteocyte cell line)	Bioprinted construct/Embedded	–	Successful 3D maturation of osteocytes with hormonal responsiveness	^177^
		In vitro	Bioink	GelMA	BMP-2	hDPSCs	Bioprinted construct/Embedded	–	Enhanced cell survival and calcified tissue growth using synthetic BMP-2	^173^
		In vitro	Bioink	ECM + AMP	–	DPSCs	Bioprinted construct/Embedded	Extrusion	∼90 % viability; AMP boosted ALP, OPN, and COL1A1 expression	^178^^,^^179^
		In vitro (human cells)	Bio-material Ink	Natural DB, 3D-printed HB	–	hSMCs, HUVECs	Printed construct/Seeded	Extrusion	Microvascular networks formation; improved scaffold cellularity with bioreactor	^185^
	Alveolar Bone	In vitro	Bio-material Ink	OsteoInk™ (HA/α-TCP)	–	Alveolar BMSCs	Printed construct/Seeded	Extrusion	High compressive strength, ISO-passed biocompatibility, precision-fit, workflow developed for clinical translation	^174^
	Perio-dontal Complex	In vitro	Bioink	GelMA + PEGDA	–	hPDLSCs	Bioprinted construct/Embedded	Inkjet	PDLSCs viability ∼82 % at 72 h (40–60 kPa); reduced spreading and viability with lower GelMA and higher PEG content	^156^
	Dentin–Pulp Complex	In vitro (human cells)	Bioink + Bio-material Ink	Gelatin + fibrinogen + HA + glycerol	–	hDPSCs	Bioprinted construct/Embedded	Extrusion	Patient-specific dentin–pulp complex; localized differentiation	^186^
		In vitro	Bioink	Alginate + dentin matrix	–	SCAPs	Bioprinted construct/Embedded	Extrusion	Cell viability >90 % at day 5 in Alg-Dent hydrogels; ALP and RUNX2 expression significantly increased by day 10	^182^
	Pulp	In vitro	Bioink	Collagen type I + agarose	–	DPSCs, HUVECs	Bioprinted construct/Embedded	Inkjet	Vasculogenesis with vascular tube formation in hydrogels	^157^
	Dentin	In vitro	Bioink	Calcium silicate + GelMA	–	SCAPs	Bioprinted construct/Embedded	Extrusion	hDPSC viability and proliferation increased with higher CS in CS/GelMA bioink; upregulated ALP, DMP-1, and OC via silicon ion release	^180^
		In vitro	Bioink	DDMp + fibrinogen + gelatin	–	DPSCs	Bioprinted construct/Embedded	Extrusion	DPSC viability >95 % in all DDMp bioinks; higher DDMp reduced proliferation but enhanced mineralization and upregulated DSPP, DMP-1 expression	^181^
		In vitro	Bioink	Poloxamer-407	–	DPSCs	Bioprinted construct/Embedded	Extrusion	EMF enhanced SCAP viability, migration, and coverage of 3D matrix; upregulated ALP, DSPP, DMP-1, and Col-1 expression	^183^
	Enamel	In vitro	Bioink	CMC + Alginate (Alg)	–	HAT-7 cells	Bioprinted construct/Embedded	Extrusion	Alg4 %-CMC2 % bioink supported HAT-7 cell viability, ALP activity, and enamel-like mineralization	^184^
	TMJ Disc	In vitro	Bio-material Ink	PCL + PEGDA	–	–	Printed construct/Acellular	Extrusion	Scaffolds showed mechanical properties closest to native TMJ disc, filament scaffolds retained modulus under hydrated conditions	^175^
		In vitro	Bio-material Ink	PLGA + PCL	CTGF, TGF-β3	Human BMSCs	Printed construct/Seeded	Extrusion	Biomimetic scaffold replicated TMJ disc structure; CTGF/TGFβ3 enhanced zonal matrix and viscoelasticity	^159^
Phase 2: Preclinical Phase (Small Animals)	Cranio-facial bone	In vivo (Mice)	Bioink	Collagen + nHAp	–	MSCs	Bioprinted construct/Embedded	Laser	Successful in situ bone regeneration	^161^
		In vitro & in vivo (mice/rats)	Bioink	Collagen + chitosan +β-glycero-phosphate + nHAp	pPDGF-B, pBMP2,	rBMSCs	Bioprinted construct/Embedded	Extrusion	Significant bone regeneration in critical-size defects (imaging-confirmed)	^187^
		In vitro & in vivo (mice)	Bioink	dECM+β TCP	None	hDPSCs	Bioprinted construct/Embedded	Extrusion	Ectopic hard tissue formation with bone/dentin markers	^188^
		In vitro & in vivo (mice)	Bioink	Collagen + chitosan +β-glycero-phosphate nHAp	rhBMP-2	rBMSCs	Bioprinted construct/Embedded	Extrusion	Enhanced bone repair with structural and genetic evidence of regeneration	^189^
		In vitro & in vivo	Bio-material Ink	Collagen type 1 + TCP	–	SCAPs	Printed construct/Seeded	Laser	Mineralized ink alone insufficient for osteogenesis; mineral phase enhanced cell migration	^196^
		In vivo (rabbit)	Bio-material Ink	6 mol% Magnesium-substituted Calcium Silicate	–	–	Printed construct/Acellular	DLP	600 μm pore size scaffold showed highest BV/TV and trabecular number; significantly better bone ingrowth and scaffold resorption compared to 480 and 720 μm designs	^162^
	Alveolar Bone	In vitro & in vivo (mice)	Bioink	MeHA + GelMA + HA	–	SVFC	Bioprinted construct/Embedded	Extrusion	Improved vascularization and bone development in implants.	^190^
		In vitro & in vivo (rats)	Bioink	GelMA + PEGDA	–	PDLSCs	Bioprinted construct/Embedded	Extrusion	4:1 GelMA/PEGDA hydrogel supported optimal PDLSC osteogenic differentiation in vitro and led to robust new alveolar bone formation in vivo	^191^
		In vitro & in vivo (rats)	Bioink	Gelatin + fibrinogen + glycerol + HA	–	hAFSCs	Bioprinted construct/Embedded	Extrusion	Functional bone and blood vessel regeneration in jaw defects	^197^
		In vitro & in vivo (rabbit)	Bio-material Ink	Silk Fibroin + Collagen + HA (SCH)	rh-EPO	–	Printed construct/Acellular (in vivo)/Seeded (in vitro)	Extrusion	Scaffold promoted osteoblast proliferation, collagen formation, and mandibular bone regeneration	^192^
		In vitro & in vivo (rabbit)	Bio-material Ink	Silk Fibroin + Collagen + nHAp	KSL-W (anti-microbial peptide), PLGA (carrier)	MC3T3-E1 (in vitro)	Printed construct/Seeded (in vitro), Acellular (in vivo)	Extrusion	Sustained antibacterial effect; excellent porosity, water absorption, biocompatibility, osteoconduction, and bone regeneration	^194^
		In vivo (mice)	Bioink	GelMA	–	HERS, DPCs	Bioprinted construct/Embedded	Extrusion	Enhanced epithelial–mesenchymal interaction (EMI), mineralized tissue formation, and significant alveolar bone regeneration over 8 weeks	^193^
	Perio-dontal Complex	In vitro & in vivo	Bioink	Collagen	FGF-2	hPDLSCs	Bioprinted construct/Embedded	Extrusion	Initial viability dip (day 1) followed by proliferative recovery (day 7) in vitro correlated with in vivo periodontal regeneration showing implant-aligned tissue with HLA/periostin/vWF/CEMP1 expression	^198^
		In vitro & in vivo	Bioink	Collagen	–	Human gingiva fibroblasts	Bioprinted construct/Embedded	Extrusion	Col/SrCS bi-layer scaffolds showed no cytotoxicity, elevated FGF-2/BMP-2/VEGF/ALP/BSP/OC in vitro, and in vivo achieved complete osteointegration versus SrCS's peripheral-only growth	^199^
	Dentin	In vitro & in vivo (mice)	Bioink	Collagen type or dECMs + β-TCP	–	DPSCs	Bioprinted construct/Embedded	Extrusion	dECM scaffolds enhanced neovascularization in vitro while maintaining strong osteogenic/dentinogenic marker expression (OPN, OCN, DSPP, DMP-1), with effects persisting for 8 weeks in vivo	^188^
	Gingiva(Oral Mucosa)	In vitro & in vivo(mice)	Bioink	Alginate + Gelatin	i-PRF	GFs	Printed construct/Seeded	Extrusion	Enhanced fibroblast viability, prolonged GF release, shaped construct formation, and angiogenesis in vivo	^200^
	TMJ Condyle	In vivo (mice)	Bio-material Ink	PCL/HA (bone phase); PGA/PLA (cartilage phase)	–	Mini-pig BMSCs, chondrocytes	Printed construct/Seeded	Extrusion	Mature osteochondral tissue with cartilage-bone interface was formed; cell sheet group showed cleaner cartilage regeneration with no residual fibers	^201^
	TMJ Disc	In vitro & in vivo(rabbit)	Bio-material Ink	PCL scaffold + PLGA microspheres	CTGF, TGFβ3	BMSCs	Printed construct/Seeded (MSC) + Bioprinted construct/Embedded (GFs)	Extrusion (multi-cartridge deposition)	Regeneration of multiphase fibrocartilage; improved healing in TMJ disc defect	^143^
		In vitro & in vivo (mice)	Bio-material Ink	PCL/PU scaffolds + dECM	PDA	Rat costal chondrocytes + L929 fibroblasts	Printed construct/Seeded	Extrusion	Enhanced ECM production, mechanical mimicry of native TMJ disc zones, and in vivo tissue regeneration in mice	^202^
	Whole Teeth	In vivo (rat)	Bio-material Ink	PU/(POSS)	Nacre	MC3T3-E1 cells	Printed construct/Seeded	Extrusion	In vitro: Good printability, mechanical strength, osteogenesis, and viability; In vivo: high bone density/volume, low resorption, no toxicity	^195^
		In vivo (rat)	Bio-material Ink	PCL + HA	SDF1, BMP7	–	Printed construct/Acellular	Not specified	Cell recruitment, periodontal regeneration, and angiogenesis confirmed by histology and quantitative analysis	^203^
Phase 3: Translational Phase (Large Animal)	Alveolar Bone	In vivo (sheep)	Bio-material Ink	HA/TCP composite	–	oAEC, oAFMSC	Printed construct/Seeded	Extrusion	Enhanced bone regeneration and vascularization in maxillary sinus defect confirmed by micro-CT and histology	^126^
	Pulp	In vivo (pig)	Bioink	GelMA microspheres	–	hDPSC-loaded microspheres	Bioprinted construct/Embedded	DLP	Promoted vascular, neural, and odontogenic tissue formation; complete dental pulp regeneration	^163^
	Tooth root, vascular pulp (Bio-root)	In vitro & In vivo (dog)	Bioink	PCL + TDM	–	DFSCs	Bioprinted construct/Embedded	Extrusion	Regenerated vascularized tooth root-like structure in vivo; favorable ECM for cell functions	^206^
	Perio-dontal Complex	In vivo (dog)	Bioink	GelMA + dECM (porcine derived)	–	DFCs	Bioprinted construct/Embedded	DLP (for PDL) DIW (for alveolar bone)	Functional regeneration of bone–ligament interface, restored PDL orientation, alveolar bone height and thickness recovery	^164^
		In vitro & In vivo (dog)	Bioink	GelMA + Sodium Alginate + BGM	BMP-2, PDGF	mBMSCs	Bioprinted construct/Embedded	Extrusion	Enhanced osteogenic and soft tissue regenerative capacity, with full periodontal tissue (bone, gingiva) regeneration	^207^
	Gingiva (Oral Mucosa)	In vivo (dog)	Bioink	ADM + Gelatin + Alginate	–	GFs	Printed construct/Seeded	Extrusion	Increased keratinized gingiva, enhanced COL I/III and VEGF-A expression	^208^
	TMJ Condyle	In vivo (pig)	Bio-material Ink	PCL	BMP-2	–	Printed construct/Acellular	Laser	Vascularized scaffold restored condylar height and anatomy with new bone formation and moderate stiffness	^204^
	TMJ Disc	In vitro & In vivo (goat)	Bio-material Ink	PCL + PVA	–	–	Printed construct/Acellular	Extrusion	Biocompatible; supported fibroblast and chondrocyte viability in vitro	^160^
									Maintained TMJ stability; protected cartilage and bone; enabled disc repair over 12 weeks in vivo	
	Whole Teeth	In vivo (dog)	Bio-material Ink	HA/PLA	–	dDPSCs	Printed construct/Seeded	Extrusion (FDM)	Enhanced mineralization observed in DPSCs-seeded scaffolds vs. cell-free controls; scaffold not fully resorbed; indicates DPSCs critical for regeneration	^205^
	Auricular and nasal cartilage	In vivo (pig)	Bio-material Ink	PCL scaffold + HA/Collagen hydrogel	–	Chondrocyte	Printed construct/Seeded	Laser	High-fidelity auricular/nasal constructs; excellent subcutaneous integration; in vitro cartilage formation in scaffold limits	^209^
Phase 4: Clinical Phase (Human)	Cranio-facial Bone	In vivo	Bio-material Ink	TCP	–	–	Printed construct/Acellular	Inkjet	Successful implantation of customized IPCAB in human patients with maxillofacial defects	^210^
		In vivo	Bio-material Ink	HA/EAM composite	–	–	Printed construct/Acellular	SLS	Custom implants matched defects; high aesthetic and functional success; 1 case of infection	^211^
	Alveolar Bone	In vivo	Bio-material Ink	Medical-grade PC08 PCL	–	–	Printed construct/Acellular	Extrusion	Volumetric bone gain, new bone formation, successful implant placement	^212^

**ADM:** Acellular dermal matrix; **AMP:** Amorphous Magnesium Phosphate; **ALP:** Alkaline Phosphatase; **BGM:** Bioactive Glass Microspheres; **BMP:** Bone Morphogenic Protein; **BMSCs:** Bone Marrow Stem Cells; **Col:** Collagen; **CMC:** Carboxymethyl chitosan; **COL1A1:** Collagen alpha-1; **CS:** Calcium Silicate; **CTGF:** Connective Tissue Growth Factor; **DB:** **Decellularized Bone; DDMp:** Demineralized Dentin Matrix Particle; **DFCs:** Dental Follicle Cells; **DIW:** Direct Ink Writing; **DMP-1:** Dentin Matrix Acidic Phosphoprotein; **DLP:** Digital Light Processing; **DPCs:** Dental Papilla Cells; **DSPP:** Dentin Sialophosphoprotein; **EAM:** Epoxide Acrylate Maleic; **ECM:** Extracellular Matrix; **EMF:** Electromagnetic Fields; **FDM:** Fused Deposition Modeling; **FGF:** Fibroblast Growth Factor; **GelMA:** Gelatin Methacryloyl; **hAFSCs:** Human Amniotic Fluid-Derived Stem Cells; **hDPSCs:** Human Dental Pulp Stem Cells; **hSMCs:** Human Smooth Muscle Cells; **HA:** Hydroxyapatite; **HAMA:** Hyaluronic Acid Methacrylate; **hPDLSCs:** Human Periodontal Ligament Stem Cells; **HB:** Hyperelastic Bone; **HERS:** Hertwig's Epithelial Root Sheath; **HGF:** Human Gingival Fibroblasts; **HUVECs:** Human Umbilical Vein Endothelial Cells; **i-PRF:** Injectable Platelet-Rich Fibrin; **IPCAB:** Inkjet-Printed Custom-Made Artificial Bones; **kCA:** Kappa Carrageenan; **LPA:** Lithium Phenyl-2,4,6-Trimethylbenzoylphosphinate; **mBMSCs:** Mouse Bone Marrow Stem Cells; **Me-HA:** Methacrylated Hyaluronic Acid; **MSCs:** Mesenchymal Stromal Cells; **nHAp:** nano-Hydroxyapatite; **nSi:** nano-Silicate; **NICE:** Nano-Engineered Ionic Covalent Entanglement; **oAEC:** Ovine-Derived Amniotic Epithelial Cells; **oAFMSC:** Ovine-Derived Amniotic Fluid Mesenchymal Stem Cells; **OPN:** Osteopontin; **PDA:** Polydopamine; **PDGF:** Platelet-Derived Growth Factor; **pPDGF-B:** Platelet-Derived Growth Factor-B Encoded Plasmid-DNA; **PCL:** Polycaprolactone; **PEGDA:** Poly(Ethylene Glycol) Diacrylate; **PLA:** Polylactic Acid; **PLGA:** Poly(D,L-Lactic-co-Glycolic Acid); **POSS:** Polyhedral Oligomeric Silsesquioxane; **PU:** Polyurethane; **PVA:** Polyvinyl Alcohol; **rBMSCs:** Rat Bone Marrow Stem Cells; **rh-EPO: Recombinant Human Erythropoietin; SCAPs:** Stem Cells from Apical Papilla; **SSCs:** Skeletal Stem Cells; **SSPCs:** Skeletal Stem and Progenitor Cells **SrCS:** Strontium-Doped Calcium Silicate; **SLS:** Selective Laser Sintering; **SVFC:** Stromal Vascular Fraction-Derived Cells; **Tb.Th:** Trabecular Thickness; **TCP:** Tricalcium Phosphate; **TDM:** Treated Dentin Matrix; **vWF:** von Willebrand Factor; **VEGF:** Vascular Endothelial Growth Factor.

#### 3.3.4 Clinical 3D bioprinting workflow

The clinical 3D bioprinting workflow begins with high-resolution medical imaging, commonly CBCT, CT, or MRI, to obtain patient-specific anatomical datasets, which are exported in the Digital Imaging and Communications in Medicine (DICOM) standard.^61^^,^^166^ These imaging datasets are subsequently processed into 3D anatomical models through computer-aided design (CAD) tools and saved in the Standard Tessellation Language (STL) file type for downstream fabrication. This digital model informs the design of a biomimetic construct that addresses both the structural and biological requirements of the defect site.^167^

The finalized design guides the development of customized bioinks, typically composed of living cells and biocompatible materials, supplemented with growth factors or other bioactive molecules. These bioinks are then deposited using inkjet, extrusion, or laser-assisted bioprinting systems selected based on construct complexity and bioink compatibility.^33^^,^^37^^,^^61^^,^^129^^,^^168^ Following fabrication, constructs undergo post-printing maturation, often in bioreactor systems, to promote cellular organization, mechanical integrity, and vascularization before clinical application.^25^^,^^61^^,^^169^

**Fig. 3:**
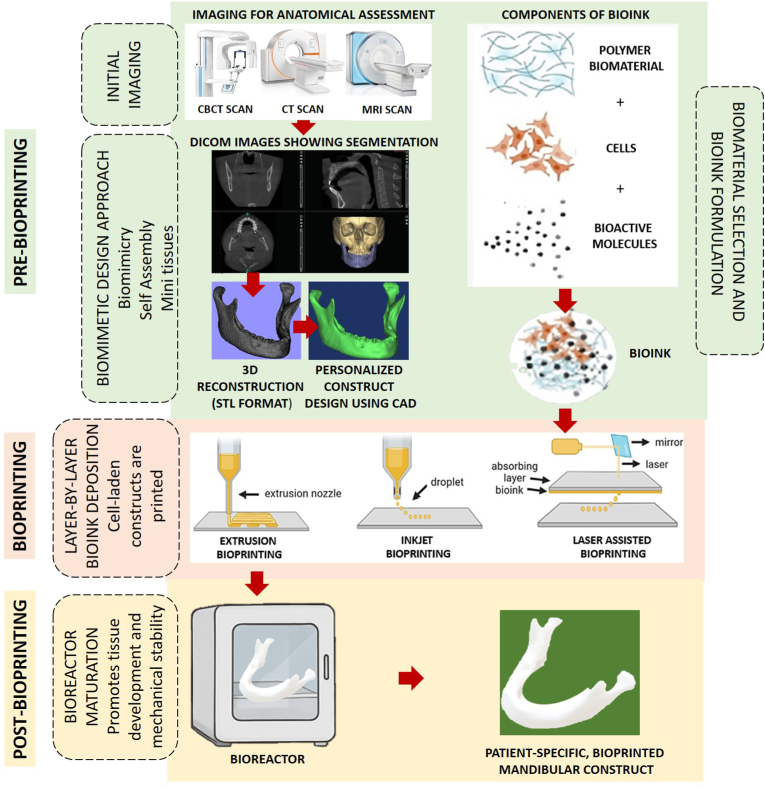
Overview of the 3D bioprinting workflow, illustrating pre-bioprinting (imaging, modeling, and bioink formulation), bioprinting (layer-by-layer deposition using different modalities), and post-bioprinting (construct maturation and functional integration). Partially adapted from ref. (33, 168) distributed under Creative Commons Attribution License (CC BY 4.0).

Although widely applicable in regenerative medicine, this workflow is examined here for its relevance to oral and maxillofacial reconstruction. It enables patient-specific construct fabrication for complex craniofacial defects and multi-tissue regeneration (Fig. 3).

## 4 Evolution of 3D bioprinting in OMF reconstruction

3D bioprinting represents a paradigm shift in OMF reconstruction, integrating computational design with biological precision to fabricate spatially organized, functional tissue constructs.^1^^,^^61^ The technique involves the layer-by-layer deposition of biological materials, biochemicals, and living cells to produce complex 3D structures with spatial control over their functional components.^2^^,^^25^^,^^37^

To ensure definitional clarity, this review adopts the consensus definition proposed by Groll et al. and Moroni et al., who describe bioprinting as “computer-aided transfer processes for patterning living and/or non-living materials into prescribed 2D/3D architectures,” encompassing both cell-laden bioinks and acellular biomaterial inks.^170^

To address methodological variability and ensure consistent study selection, we employ a dual-axis classification system based on: (1) Material type, distinguishing between bioinks (cell- or bioactive molecule-containing formulations) and biomaterial inks (acellular structural materials)^58^; and (2) Fabrication approach, classifying constructs as either (i) bioprinted, in which cells are embedded during fabrication (meeting ASTM criteria for true bioprinting with in-process cell patterning),^171^ or (ii) printed, where scaffolds are seeded post-fabrication or used acellularly, as in traditional 3D printing.^172^ The translational evolution of 3D bioprinting in OMF reconstruction can be mapped into four functional phases, based on increasing biological complexity and anatomical relevance:

### 4.1 Phase 1: Laboratory phase (foundational work)

Initial efforts focused on in vitro validation of bioprinted constructs. Bioinks such as alginate–gelatin–nano-hydroxyapatite (nHAp) hydrogels supported >90 % viability of human periodontal ligament stem cells (hPDLSCs) and promoted osteogenic differentiation via alkaline phosphatase (ALP) activity.^158^ Gelatin methacryloyl (GelMA) bioinks embedded with dental pulp stem cells (DPSCs) facilitated calcified tissue formation in the presence of synthetic BMP-2.^173^ Acellular biomaterial inks, such as polycaprolactone (PCL) and hydroxyapatite (HA), were frequently printed and manually seeded to approximate periodontal and alveolar bone architecture.^174^ Studies have also explored PLGA–PCL scaffolds and PCL–PEGDA composites for TMJ disc engineering.^159^^,^^175^ These studies established biocompatibility and print fidelity, though functional integration and vascularization remained absent. Representative in vitro studies supporting Phase 1 findings are summarized in Table 4.^156^, ^157^, ^158^, ^159^^,^^173^, ^174^, ^175^, ^176^, ^177^, ^178^, ^179^, ^180^, ^181^, ^182^, ^183^, ^184^, ^185^, ^186^.

### 4.2 Phase 2: Preclinical phase (functional validation in small animals)

This phase advanced to in vivo validation using small-animal models, emphasizing biological function. Bioinks evolved to include multi-material formulations; for example, collagen–chitosan–β-tricalcium phosphate (β-TCP) composites embedded with rat bone marrow stromal cells (rBMSCs) and plasmid DNA (pBMP-2/pPDGF-B) regenerated critical-size calvarial defects in rats.^161^^,^^187^, ^188^, ^189^ Methacrylated hyaluronic acid (MeHA)–GelMA bioinks combined with stromal vascular fraction cells (SVFCs) promoted both osteogenesis and neovascularization in murine alveolar defects.^190^, ^191^, ^192^, ^193^, ^194^ Likewise, nacre-reinforced polyurethane (PU/POSS) scaffolds seeded with MC3T3-E1 cells mitigated alveolar bone resorption.^195^ Despite improved outcomes, limitations such as mismatched degradation rates and insufficient mechanical strength persisted. Key preclinical studies validating Phase 2 outcomes are summarized in Table 4.^143^^,^^161^^,^^162^^,^^187^, ^188^, ^189^, ^190^, ^191^, ^192^, ^193^, ^194^, ^195^, ^196^, ^197^, ^198^, ^199^, ^200^, ^201^, ^202^, ^203^.

### 4.3 Phase 3: Translational phase (large-animal models)

This phase introduced anatomically scaled models to bridge preclinical validation and clinical application. Regulatory-friendly biomaterial inks were commonly used. For example, in a porcine jaw model, BMP-2–incorporated PCL scaffolds restored condylar height and stimulated vascularized bone formation.^204^ Hydroxyapatite/polylactic acid (HA/PLA) scaffolds seeded with dog-derived DPSCs improved mineral deposition in alveolar reconstructions.^205^ Biomaterial inks such as hydroxyapatite/β-tricalcium phosphate (HA/β-TCP) composites have promoted host cell recruitment and tissue integration in sheep maxillary sinus models.^126^ Other large-animal studies have used GelMA microspheres for pulp regeneration,^163^ PCL with treated dentin matrix for vascularized bio-root formation,^206^ and GelMA–dECM bioinks to regenerate periodontal bone–ligament interfaces.^164^ Gingival tissue engineering has also been achieved using composite bioinks,^207^^,^^208^ while PCL–HA/collagen hybrids have been tested for auricular and nasal cartilage repair.^209^ However, long-term scaffold degradation, immune response, and load-bearing limitations remain key translational barriers. Large-animal studies bridging preclinical and clinical applications are summarized in Table 4.^126^^,^^160^^,^^163^^,^^164^^,^^204^, ^205^, ^206^, ^207^, ^208^, ^209^.

### 4.4 Phase 4: Clinical translational phase (early human applications)

This phase marks the initial clinical use of 3D-printed constructs in patients. To date, only acellular biomaterial inks have been clinically deployed. Saijo et al.^210^ used inkjet-printed custom-made artificial bone (IPCAB) for mandibular reconstruction, achieving satisfactory integration and clinical outcomes. Similarly, Zhang et al.^211^ employed selective laser sintering (SLS) to produce HA/EAM constructs for maxillofacial reconstruction, achieving 90 % postoperative patient satisfaction. Although these constructs lacked cells or bioactive factors, they represent important milestones in the clinical adoption of 3D printing for patient-specific craniofacial repair. Clinical pilot studies and early translational applications are summarized in Table 4.^210^, ^211^, ^212^.

The clinical translation of viable, cell-laden constructs remains unrealized. A scoping review by Briones et al.^213^ identified only 11 bioprinting-related clinical trials registered globally between 2016 and 2023. Of these, just four involved human implantations—and none targeted oral or maxillofacial tissues. This underscores the translational gap in Phase 4 and highlights the persistent absence of clinically viable, cell-laden constructs in OMF applications. Table 4 summarizes key studies illustrating the staged evolution of 3D bioprinting in OMF reconstruction.

## 5 Applications of 3D bioprinting techniques for OMF reconstruction

### 5.1 Anatomical and functional complexity in the OMF region

A central clinical challenge in OMF reconstruction is the repair of composite defects comprising multiple functionally distinct tissues—each requiring precise spatial organization and biomechanical integration to replicate native anatomy and function.^1^^,^^2^^,^^172^ The OMF region includes structurally diverse tissues ranging from soft, vascularized dental pulp to highly mineralized enamel and alveolar bone.^33^^,^^90^^,^^214^ These tissues differ substantially in architecture (e.g., tubular, porous, anisotropic), mechanical properties (from viscoelastic to rigid), and biological roles (e.g., load-bearing, nutrient exchange, or signaling).^77^^,^^179^^,^^215^ As such, a uniform bioprinting strategy is inadequate. Successful reconstruction requires fine-tuned customization across bioink composition, scaffold mechanics, and spatial resolution.^2^^,^^37^^,^^61^

While Table 4 illustrates the staged translational progression of 3D bioprinting in OMF reconstruction, the following subsections examine how specific tissue-targeted strategies address structural and biological demands. Key material and design parameters are summarized in Table 5.^216^

**Table 5:** Requirements for bioinks in OMF reconstruction.

Tissue Type	Structural Requirements	Mechanical Requirements	Biological Requirements	Additional Considerations	References
**Enamel**	- Highly mineralized, acellular structure composed of hydroxyapatite - Prismatic rod arrangement - No ECM or cellular component	-Extremely high hardness (∼5 GPa) - Young's modulus ≈ 84 GPa - Brittle, with low tensile strength	-No endogenous cells; cannot regenerate naturally - No reparative ability	-Requires biomimetic or synthetic substitution - Must resist mechanical wear, chemical erosion, and bacterial activity - Multilayered composite strategies may be needed	^184^^,^^221^
**Dentin**	-Tubular mineralized structure - Microporosity ≈ 300 μm - Hydroxyapatite framework	-Young's modulus: 17 GPa (pulp side) to 42 GPa (center)	-Stimulate odontoblast activity - Allow cell extension and matrix deposition	-Pore architecture critical for regeneration	^90^^,^^172^^,^^182^^,^^214^^,^^221^
**Dental Pulp**	-Zonal architecture: cell-rich core, cell-free zone, odontoblast layer - Collagen-based ECM - Prefer hydrogel scaffolds	- Storage modulus ≈ 100 Pa - Loss modulus ≈ 10 Pa - Young's modulus ≈ 0.8 ± 0.4 kPa	- Maintain DPSC viability and proliferation - Support differentiation near dentin interface	- Mimic viscoelasticity for stress relaxation and nutrient diffusion - Heterogeneity is key	^90^^,^^172^^,^^215^
**PDL**	- Collagen-rich, vascularized connective tissue - ECM with dynamic load response	- Young's modulus ≈ 5 × 10^6^ N/m^2^ - Poisson's ratio = 0.45 - Stiffness: 6–135 kPa - Time-dependent viscoelasticity	- Support fibroblasts, PDLSCs, osteoblasts, cementoblasts - Enhance osteogenic potential	- Stiffness affects stem cell behavior - Models should consider force relaxation and hysteresis	^1^^,^^90^^,^^172^^,^^222^
**Cranio-facial/Alveolar Bone**	- Multi-layered architecture: periosteum, compact/cancellous bone, cribriform plate - Pore size: 150–500 μm	- Young's modulus: 0.9 × 10^9^ N/m^2^ (cancellous) to 13.7 × 10^9^ N/m^2^ (compact)	- Support osteoblasts, osteoclasts, xosteocytes - Enable mineralization with HA + collagen type I	- Composite materials may be needed - Vascularization essential for scaffold integration	^1^^,^^90^^,^^172^^,^^187^
**TMJ Disc**	- Fibrocartilaginous, biconcave structure with collagen zones - Anisotropic orientation critical	- Compressive modulus: 0.2–3.0 MPa - Tensile modulus: ∼25–52 MPa	- Support fibrocartilage phenotype - Withstand compression and shear	- High tensile/compressive strength (0.01–0.05 GPa) per stress cycles	^1^^,^^2^^,^^77^^,^^159^^,^^160^
**Gingiva/Oral Mucosa**	- Keratinized gingiva: Orthokeratinized epithelium, dense collagen matrix - Oral mucosa: Non-keratinized epithelium, elastin-rich connective tissue	- Tensile strength: 1.06–3.94 MPa (gingiva > mucosa) - Young's modulus: 2.48–19.75 MPa (region-dependent) - Viscoelasticity: Stress relaxation 48–59 % (mucosa > gingiva) - Compressive resistance: Attached gingiva > mucosa (peak stress 0.2–1.17 MPa)	- Keratinocyte proliferation (barrier function) - Fibroblast activity for ECM remodeling - Collagen/elastin balance for tissue flexibility	- Dynamic loading adaptation (mastication forces) - Hydration maintenance critical	^223^^,225^
**Whole Tooth**	- Multi-tissue: enamel, dentin, pulp, PDL, cementum - Tissue interfaces and orientation are essential	- Gradient stiffness: Enamel ∼84 GPa; Dentin ∼17–42 GPa; PDL ∼0.1 MPa	- Coordinate multiple cell populations for functional integration	- Requires synchronized multi-material, zonal printing strategy	^1^^,^^195^^,^^203^^,^^221^

**ECM:** Extracellular Matrix; **DPSC:** Dental Pulp Stem Cells; **PDLSC:** Periodontal Ligament Stem Cells; **PDL:** Periodontal Ligament; **TMJ:** Temporomandibular Joint; **HA:** Hydroxyapatite; **Mpa:** Megapascal; **Gpa:** Gigapascal.

### 5.2 Design optimization based on target tissue type

#### 5.2.1 Soft tissues: connective and vascularized components

To replicate the pulp's viscoelastic and angiogenic microenvironment, soft hydrogels such as gelatin, fibrinogen, and hydroxyapatite have been used in bioinks. Human dental pulp stem cells (hDPSCs), capable of both odontogenic and vasculogenic differentiation, are commonly employed. Han et al.^186^ engineered bilayer constructs with region-specific stiffness, promoting localized mineralization and vascularized soft tissue formation. More recently, Qian et al.^163^ used GelMA microsphere-based bioinks with digital light processing (DLP) to regenerate vascularized and innervated pulp tissue in a porcine model.

For oral mucosa regeneration, customization focuses on rapid gelation, elasticity, and fibroblast viability. Yi et al.^200^ demonstrated that i-PRF–enhanced collagen/alginate constructs seeded with human gingival fibroblasts enhanced angiogenesis, keratinized tissue formation, and epithelial integration. Similarly, Wang et al.^199^ achieved full osteointegration and epithelial regeneration using collagen/SrCS bilayer scaffolds.

#### 5.2.2 Hard tissues: mineralized and load-bearing structures

Bioprinting enamel is particularly challenging due to its acellular, highly mineralized composition and exceptional stiffness (∼84 GPa).^90^ Mohabatpour et al.^184^ developed extrusion-bioprinted 4 % alginate/2 % CMC scaffolds seeded with ameloblast-like HAT-7 cells, which maintained viability, enhanced ALP expression, and promoted enamel-like mineralization with geometric precision.

Dentin regeneration involves bioinks loaded with calcium-based fillers (e.g., calcium silicate, dentin matrix particles) and cells such as DPSCs or SCAPs with odontogenic potential. Lin et al.^180^ reported enhanced proliferation and odontogenic marker expression (ALP, DSPP, DMP-1) using SCAPs embedded in calcium silicate–GelMA constructs. Han et al.^181^ showed that higher concentrations of decellularized dentin matrix improved mineralization, despite reduced proliferation—highlighting the trade-off between scaffold bioactivity and cell expansion.

Alveolar bone regeneration requires osteoconductive, vascularizable, and mechanically robust materials. Composite bioinks comprising GelMA, HA, and silk fibroin are commonly combined with MSCs or BMSCs to replicate cortical and cancellous bone layers. Anderson et al.^174^ introduced OsteoInk™, a HA/α-TCP composite with high compressive strength and ISO-certified biocompatibility. In vivo, Liu et al.^192^ and Li et al.^194^ demonstrated that silk fibroin–collagen scaffolds enhanced osteogenesis, osteoblast proliferation, and antibacterial performance. Both extrusion and DLP systems enabled anatomically precise, load-bearing scaffold fabrication.

#### 5.2.3 Multi-tissue constructs: hybrid and zonal interfaces

The PDL complex, encompassing the ligament, cementum, and adjacent alveolar bone, requires spatially organized, multi-tissue regeneration.^33^ Customization strategies emphasize scaffold stiffness, architecture, and cell patterning to recreate this biomechanical interface.^1^^,^^90^ GelMA–PEGDA composites tuned to match PDL mechanical properties (6–135 kPa) were seeded with hPDLSCs to support fibroblastic, cementogenic, and osteogenic differentiation.^90^^,^^172^ Ma et al.^156^ showed that inkjet-printed scaffolds maintained PDLSC viability and morphology with optimized mechanics.

In vivo, Lee et al.^198^ used collagen-based scaffolds seeded with hPDLSCs and FGF-2 to regenerate aligned fibers and facilitate cementum–bone integration, as confirmed by periostin, vWF, and CEMP1 expression. Yang et al.^164^ further advanced this strategy using a dual-mode platform, DLP for PDL and direct-ink-writing (DIW) for alveolar bone, which successfully re-established the ligament–bone interface and restored alveolar bone volume in large animals.

TMJ reconstruction requires dual-regeneration of the fibrocartilaginous disc and osteochondral condyle.^77^ PLGA- or PCL–PEGDA–based scaffolds seeded with BMSCs, and stimulated with CTGF and TGF-β3, have demonstrated zonal matrix production and biomechanical fidelity.^159^^,^^175^ Simultaneously, biphasic PCL/HA–PGA/PLA scaffolds co-seeded with mini-pig BMSCs and chondrocytes supported osteochondral interface formation and restored condylar height in vivo.^201^ Together, these strategies demonstrate integrated solutions for functional TMJ regeneration.

Full-tooth regeneration requires spatial coordination of enamel, dentin, pulp, cementum, and PDL layers. Multi-material scaffolds and co-culture systems are used to replicate this complexity. Gong et al.^195^ employed PU/POSS-nacre composites seeded with MC3T3-E1 cells to achieve mineralized tissue and periodontal regeneration. Chen et al.^205^ used HA/PLA scaffolds with DPSCs to support mineralization. Huang et al.^206^ reconstructed bio-root structures using PCL and treated dentin matrix seeded with DFSCs, demonstrating the translational potential of hybrid extrusion systems.

## 6 Translational barriers in clinical 3D bioprinting for OMF reconstruction

**Fig. 4:**
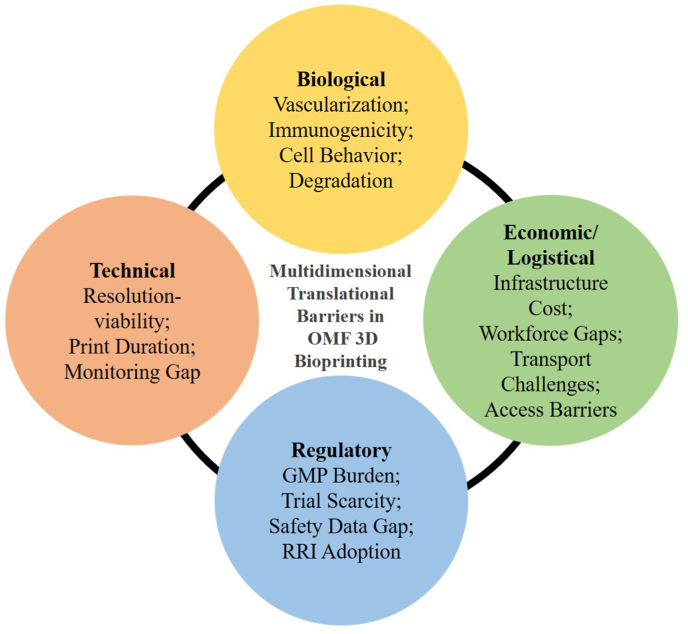
Schematic summary of the four major translational challenge domains in 3D bioprinting for OMF reconstruction. Each circle highlights key representative barriers encompassing biological, technical, regulatory, and economic/logistical dimensions.

Despite its transformative promise, the clinical translation of 3D bioprinting in oral and maxillofacial (OMF) reconstruction remains constrained by four primary categories of barriers: biological, technical, regulatory, and economic/logistical. These interrelated challenges are summarized in Fig. 4.

### 6.1 Biological barriers

Key biological limitations include: (i) insufficient vascular networks, which restrict nutrient diffusion and prevent viability in constructs exceeding 300 μm in thickness; (ii) neural integration strategies that remain in preclinical or experimental stages, with no validated protocols for clinical application,^39^ (iii) unpredictable stem cell responses to mechanical and thermal stresses imposed during bioprinting,^1^ and (iv) poorly characterized immunogenicity of certain bioink components, which may provoke adverse host responses.^35^^,^^171^

### 6.2 Technical limitations

Technical barriers stem from core manufacturing and process control limitations: (i) the inherent trade-off between resolution and cell viability in extrusion-based bioprinting^171^; (ii) limited scalability, as prolonged printing durations can compromise cell viability and structural fidelity; (iii) the absence of integrated real-time quality control systems during fabrication; and (iv) a lack of standardized, open-access bioink databases to support reproducibility and protocol optimization.^35^

### 6.3 Regulatory hurdles

The regulatory landscape for bioprinted constructs remains uncertain and highly restrictive: (i) Classification as Advanced Therapy Medicinal Products (ATMPs) or Human Cells, Tissues, and Cellular- and Tissue-Based Products (HCT/Ps) requires adherence to GMP regulatory guidelines^171^; however, successful approvals remain rare—few cell and gene therapies have received FDA clearance, and the European Medicines Agency (EMA) has authorized a limited number of ATMPs, reflecting high regulatory thresholds^217^; (ii) while acellular constructs represent regulatory progress, cell-laden OMF bioprints have yet to enter human trials,^1^^,^^39^ (iii) long-term safety data regarding immune response, ectopic tissue formation, and scaffold degradation are lacking^59^; and (iv) Responsible Research and Innovation (RRI) frameworks remain underutilized, limiting societal engagement and ethical foresight.^171^

### 6.4 Economic and logistical constraints

Practical implementation is further hindered by significant economic and logistical challenges: (i) GMP-compliant bioprinting facilities require capital investments ranging from $2–5 million^35^; (ii) interdisciplinary workforce shortages persist, particularly in clinicians and engineers trained in bioprinting technologies^35^^,^^61^; (iii) transportation of living bioprinted constructs presents unresolved logistical issues, including temperature control and sterility^39^; and (iv) reimbursement frameworks for bioprinted implants are currently nonexistent, limiting institutional adoption and scalability.^35^^,^^59^

## 7 Future perspectives in clinical bioprinting for OMF reconstruction

Advances in 3D bioprinting are progressively shifting the field from proof-of-concept research to clinically adaptable solutions. Next-generation platforms—such as 4D bioprinting, vascular–neural integration, organoid-based systems, and AI-enabled automation—hold promise for overcoming persistent barriers in OMF tissue engineering.

### 7.1 Integration of 4D bioprinting and smart biomaterials

4D bioprinting introduces dynamic constructs capable of transforming in response to physiological stimuli. Smart biomaterials, including shape memory polymers and thermoresponsive hydrogels, allow constructs to alter their form or mechanical characteristics in response to environmental factors like thermal fluctuations, pH shifts, and changes in ambient moisture.^165^^,^^218^ For example, biodegradable shape-memory materials like chitosan–PCL blends support staged tissue recovery aligned with remodeling phases. However, technical challenges remain, particularly in achieving controlled actuation, long-term mechanical stability, and consistent in vivo performance.^2^

### 7.2 Vascularization and neuro-regeneration strategies

Long-term success in OMF bioprinting requires the integration of pre-vascularized and innervated constructs to ensure nutrient exchange, functional responsiveness, and biological viability. Vascularization approaches include the use of endothelial cell–laden bioinks, controlled VEGF release, and sacrificial templating techniques to promote host vessel ingrowth.^2^^,^^61^ In parallel, neural regeneration strategies employ neurotrophic factor-enriched scaffolds, axon-guiding topographies, and conductive hydrogels to facilitate functional neural interface development.^219^ Despite these advances, coordinated neurovascular maturation remains a major translational hurdle.

### 7.3 Organoid and spheroid-based bioprinting

Organoid and spheroid-based bioprinting offers scaffold-free, self-organizing constructs with high biomimicry. Organoids derived from stem cells can recapitulate complex OMF structures such as salivary glands and tooth germs, while cell spheroids facilitate tissue fusion and vascularization.^220^ Nonetheless, clinical translation is hindered by issues such as structural fragility, size heterogeneity, and poor integration with vascular networks. Incorporating vascularized organoids and applying bioreactor-based maturation protocols may enhance construct viability and scalability.^2^^,^^61^

### 7.4 AI and automation in bioprinting

Artificial intelligence (AI), machine learning (ML), and robotic systems are increasingly being integrated into bioprinting workflows to improve automation, precision, and reproducibility. AI tools aid in image segmentation, defect-specific modeling, and real-time parameter optimization. Robotic platforms enhance in situ bioprinting accuracy, while ML algorithms are used to predict bioink behavior and optimize printing conditions. Closed-loop systems that combine AI with real-time sensors and adaptive control mechanisms are emerging as critical enablers of scalable, patient-specific fabrication.^221^These automated and robotic platforms provide the technological foundation needed to progress toward intraoperative, in situ bioprinting strategies.

### 7.5 In situ and intraoperative bioprinting

In situ or intraoperative bioprinting—where bioinks are deposited directly into the surgical defect using handheld or robotic devices—represents a major future translational milestone for OMF surgery.^221^ To date, no study has reported its specific application in OMF reconstruction, positioning it as a compelling direction for future research. However, to the best of the authors’ knowledge, only isolated work exists in related OMF tissues, for example, a handheld bioprinting approach enabling vasculogenesis within dental pulp spaces^222^ and a recent review summarizing portable hand-held in situ bioprinting platforms across multiple tissues,^223^ but none demonstrating true OMF defect reconstruction.

This approach could address key translational bottlenecks by enabling single-stage filling of complex craniofacial defects (e.g., alveolar ridge deficiencies, post-resection cavities) with constructs that closely match the patient's anatomy. Realizing this potential will require overcoming OMF-specific challenges, including: (1) designing bioinks that solidify rapidly and remain stable in a saliva-moistened, dynamic environment; (2) developing ergonomic, sterile delivery systems—either handheld or image-guided robotic; and (3) integrating bioprinting with intraoperative imaging modalities such as cone-beam CT to ensure precise deposition. Focused preclinical work in anatomically relevant OMF models represents the next essential step toward evaluating the feasibility of this paradigm.

## 8 Discussion

The clinical translation of 3D bioprinting for oral and maxillofacial (OMF) reconstruction reflects a dynamic interplay between biological feasibility, technological innovation, and clinical applicability. As outlined in this review, early laboratory work (Phase 1) established foundational principles using stem cells and hydrogels with demonstrated viability and osteogenic potential.^141^^,^^158^^,^^159^ These findings were expanded in small-animal models (Phase 2), where pre-vascularization strategies and multi-material constructs achieved promising regenerative outcomes.^187^^,^^190^^,^^195^ Subsequent large-animal studies (Phase 3) validated anatomical relevance but relied primarily on acellular biomaterial inks, reflecting regulatory caution and the need for scaffold standardization.^164^^,^^204^^,^^205^ Despite successful deployment of printed, patient-specific constructs in humans (Phase 4), none have yet employed viable, cell-laden bioinks—underscoring a persistent translational gap.^210^^,^^211^^,^^213^

Customization remains a core requirement for clinical success in OMF bioprinting, given the region's functional diversity and spatial constraints.^1^^,^^61^^,^^172^ Soft tissue targets such as dental pulp and gingiva demand angiogenic compliant matrices,^163^^,^^186^^,^^199^^,^^200^ while mineralized tissues like enamel, dentin, and alveolar bone require mechanically robust, osteoconductive scaffolds.^174^^,^^180^^,^^184^^,^^192^ Interfaces such as the periodontal ligament and temporomandibular joint introduce additional challenges in stiffness gradients and multi-tissue coordination.^156^^,^^159^^,^^198^ Current strategies using multi-material bioinks, zonal architectures, and dual-mode bioprinting platforms have shown promise in reconstructing these complex anatomical units, though most remain at the preclinical stage.^164^^,^^201^

As reviewed in Section 5, major translational barriers persist across biological, technical, regulatory, and economic dimensions. Vascularization and innervation remain critical for achieving functional thickness and integration,^2^^,^^39^^,^^62^ while technical constraints—such as resolution-viability tradeoffs and quality control limitations—impact reproducibility and scalability.^35^^,^^171^ Regulatory frameworks for cell-laden constructs remain fragmented, with most approvals limited to acellular devices,^1^^,^^171^^,^^217^ and economic barriers such as GMP facility costs and lack of reimbursement pathways further hinder clinical rollout.^35^

Emerging platforms such as 4D bioprinting, organoid- and spheroid-based strategies, and AI-driven automation offer meaningful paths forward.^2^,^218^,^220^,^224^ However, these technologies must be aligned with clinically validated workflows and supported by robust interdisciplinary collaboration. Continued progress will depend on harmonizing innovation with safety, reproducibility, and clinical relevance to enable the translation of 3D bioprinted constructs from laboratory prototypes to viable therapeutic solutions.

## 9 Conclusion

Over the past decade, 3D bioprinting has progressed from a conceptual innovation to a transformative strategy for oral and maxillofacial reconstruction, propelled by advances in bioink formulation, scaffold design, and multi-tissue integration. Despite this progress, clinical translation remains constrained by persistent challenges, notably inadequate vascularization and innervation, lack of manufacturing standardization, and limited long-term functional validation. Next-generation approaches—such as dynamic bioprinting techniques, organoid-inspired constructs, neurovascular patterning, and AI-guided fabrication—show promise for tackling these challenges. Turning these innovations into clinical reality will require continuous interdisciplinary efforts spanning engineering, biology, and regulatory expertise to deliver patient-specific solutions for complex OMF defects.

## Author contribution

Conceptualization, S.D.; M.A.S.; G.K. and D.R.; methodology, S.D.; M.A.S.; G.K. and D.R.; validation, S.D.; M.A.S.; G.K. and D.R.; formal analysis, S.D.; G.K. and D.R.; investigation, S.D.; M.A.S.; G.K. and D.R.; writing—original draft preparation, S.D.; M.A.S.; G.K. and D.R.; writing—review and editing, S.D.; M.A.S.; G.K. and D.R.; visualization, S.D.; M.A.S.; G.K. and D.R.; supervision, S.D.; M.A.S.; G.K. and D.R.; funding acquisition, S.D.; M.A.S.; G.K. and D.R. All authors have read and agreed to the published version of the manuscript.

## Consent

Not applicable.

## Ethical clearance

Not applicable.

## Funding

None.

## Declaration of competing interest

The authors declare that they have no known competing financial interests or personal relationships that could have appeared to influence the work reported in this paper.

## Data Availability

The authors confirm that the data supporting the findings of this study are available within the article and if any remaining/additional data is required, it will be provided by the corresponding author on reasonable request.
